# Pilot study results from the assertive community treatment transitional intervention program in Switzerland

**DOI:** 10.1038/s41598-025-93470-y

**Published:** 2025-03-15

**Authors:** Mariela E. Jaffé, Julian Moeller, Franziska Rabenschlag, Christine Althaus Aebersold, Jörg Eysell, Constantin Bruttel, Lukas Imfeld, André Nienaber, Undine E. Lang, Christian G. Huber

**Affiliations:** 1 Universitäre Psychiatrische Kliniken (UPK) Basel, University of Basel, Wilhelm Klein-Str. 27, 4002 Basel, Switzerland; 2 Center of Social Psychology, Faculty of Psychology, University of Basel, Basel, Switzerland; 3 Division of Clinical Psychology and Epidemiology, Faculty of Psychology, University of Basel, Basel, Switzerland; 4 ZI Mannheim, Mannheim, Germany

**Keywords:** Assertive community treatment, Transitional intervention, Relapse reduction, Pilot study, Psychology, Health care

## Abstract

Often, after an inpatient stay, early readmission occurs, which is detrimental to the patient. University Psychiatric Clinics Basel developed a 3-month transitional intervention program to bridge inpatient and community treatment by supporting people after their discharge from the psychiatric hospital. In line with international guidelines, this transitional intervention was delivered as assertive community treatment in the homes of people who wished to participate. Data from the 3-year pilot project starting in 2019 have been collected to analyze the success of its implementation and treatment effectiveness in reducing follow-up inpatient treatment days when comparing people choosing to participate in the transitional program and receiving treatment (*n*_*cases*_ = 456) versus people declining participation (*n*_*cases*_ = 104). Results indicate that within 3 years, a multidisciplinary team could be assembled that was able to work with a caseload of up to 66 persons per month. Receipt of treatment was descriptively associated with lower numbers of inpatient treatment days, stays, and involuntary admissions 6 months after discharge; however, this difference did not reach statistical significance. Program participants further reported very high levels of satisfaction. These findings provide an outlook on the program’s feasibility and potential benefits.

## Introduction

Previous research describes a substantial risk that people with mental health problems might be readmitted to inpatient care within the first few months after being discharged^1–3^. The change from a highly structured inpatient setting where support is available almost 24 h a day to being at home with only some form of outpatient care, such as regular visits to a psychiatrist, clinical psychologist or mental health specialist, is substantial^2^—and this is especially true for people with severe mental illnesses, where their mental, behavioral, or emotional disorder results in serious functional impairment^4^. Different measures can be taken to attenuate the magnitude of this change. For instance, pre-discharge procedures could include therapeutic leave^5,6^ or the scheduling of follow-up outpatient appointments during the inpatient stay. Post-discharge measures may entail telephone follow-ups. Bridging measures already start during an inpatient stay and continue beyond it, where transition managers may be appointed who support people before, after or during that change (for a review, see^3^). Given the severity of early relapses and the positive effect sizes reported for transitional interventions^3,7^, University Psychiatric Clinics (UPK) Basel developed a program to bridge inpatient and outpatient mental health care taking place in individuals’ local community. Transitional assertive community treatment was available for all patients that may benefit from support after an inpatient stay. This transitional intervention is set up during the inpatient stay and continues for up to three months after discharge^1,2^. The clinic’s newly developed program was implemented as a 3-year pilot study in 2019 and we here present the initial results on its feasibility and implementation as well as on outcomes regarding readmission rates and participants’ satisfaction.

## Background

Assertive community treatment^8^ is recommended by international guidelines (e.g., S3-Guidelines by the DGPPN^9^ or the NICE-Guideline on psychosis and schizophrenia in adults^10^) and multiple studies have highlighted its effectiveness in supporting people with severe mental illnesses^11^. These continuous treatment programs are usually delivered by a multidisciplinary team, designed to meet the specific requirements of the person, and often provide open-ended support. Nevertheless, there are different opinions on whether an unlimited treatment timeframe is a critical component of assertive community treatment^12^. Shorter interventions also seem to be effective^1,13,14^, and as early readmissions (often defined as a readmission within 90 days of discharge; see^3^) appear especially destabilizing, it seems important to provide a treatment offering a bridge for these first three months after discharge.

In Switzerland, however, assertive community treatment or outreach mental health treatment is not yet widely available in routine mental health care as funding through basic health insurance has, so far, not been established^15,16^. Instead, mental health care treatment is offered in general in inpatient and outpatient settings. For the canton of Basel-Stadt, for example, UPK Basel serves as a large public psychiatric hospital that provides inpatient psychiatric services to the surrounding population (approximately 200,000 individuals). Furthermore, day clinic services and outpatient care are available at UPK Basel. The outpatient mental health care landscape is further complemented by other institutions’ outpatient or day clinics and by an independent network of psychiatrists and psychologists^17^.

Traditionally, the change from an inpatient to outpatient setting was part of the discharge plan developed during an inpatient or day clinic stay at UPK Basel. This was organized within each unit and no clinic-wide standardized concept was present. The objective of the discharge plan was to secure an outpatient option that would allow continuous mental health treatment. However, after discharge from the clinic, there was no opportunity to monitor whether outpatient treatment was utilized or support for further uptake of therapy. Discontinuation of treatment was therefore possible and support during the critical period of changing from inpatient to outpatient settings limited.

Assertive community treatment—transitional intervention (ACTTIV) by UPK Basel targets exactly this problem. Complementing long-term recovery-oriented assertive community treatment (Re-ACT; see^18^), which was developed in parallel, ACTTIV offers assertive community treatment to all individuals 18 years or older and-irrespective of the clinical diagnoses-for a transition period of 3 months after discharge from an inpatient stay. It aims to facilitate the transition from an inpatient to community-based living arrangement by providing tailored support in individuals’ homes or community (depending on their preference). If the organization of outpatient support was part of the agreed therapeutic goals, ACTTIV team members would support people in this too.

As the ACTTIV program was newly introduced in 2019 as a pilot project, it was accompanied by a continuous evaluation to ensure that the implementation would be feasible not only as a pilot project but also as a regular treatment offered by the clinic. Furthermore, the evaluation aimed to test whether the program is effective in reducing early and eventually even involuntary readmissions to inpatient care in the first months after discharge. We here present results from a 3-year pilot phase looking at (a) the composition of the project team and case numbers over the 3-year period, (b) the participants joining the ACTTIV program versus declining participation, (c) their objective outcome measures such as number of inpatient treatment days and (d) the subjective outcome measure of participants’ satisfaction with the program.

## Method

### Assertive community treatment—transitional intervention (ACTTIV) in Basel

The ACTTIV program was designed to support people during the first 90 days of community living after being discharged from a psychiatric inpatient stay at the UPK Basel. All persons 18 years or older (irrespective of their diagnoses) were eligible to join.

The ACTTIV team has a multidisciplinary background and includes psychiatrists, social workers and nurses. After initial contact during the inpatient stay, the therapeutic objectives were agreed upon during the first sessions. Objectives always included self-determination and self-empowerment in the person’s life. Topics that may be included are handling of mental health problems and symptoms, forming social connections, relationships, family, and sexuality, living, daily activities, work, leisure, administration, and finances. Individual needs^19^ were first assessed and the ACTTIV program was then tailored to the needs that the program participant and project team decided were the most important. The team valued including family members, friends, and employers and, if participants agreed, would integrate them into the transitional intervention. The program’s office hours covered all workdays (Monday to Friday) from 8 am to 5 pm. Participation costs of the program were covered by the general health insurance and the canton of Basel-Stadt.

Individual meetings either take place in the participant’s home or, if they prefer not to meet at home, in their community (e.g., a park or a café). The frequency of visits depends on patient needs and therapy goals throughout the transition process after the inpatient stay. Nurses can provide up to 4 h of treatment and services per week. In crises, psychiatrists in the team can provide further time and treatment for the participant.

The ACTTIV program started in April 2019 and a 3-year pilot phase was scheduled until March 2022. Parallel to this period, the project’s implementation and first outcomes were continuously evaluated. Swissethics / Ethikkommission der Nordwest- und Zentralschweiz (EKNZ) provided ethics approval for this procedure (ID 2019-00531). Informed consent was collected from participants for a different part of the research project. For the here presented results, which stem from clinical routine data, however, the requirement to collect informed consent was waived by the ethics committee. All procedures were executed in line with the Declaration of Helsinki in its latest revision. With the approval of the local ethics committee, anonymized clinical data from inpatients of the psychiatric clinic was used to create a comparison group of persons that did not join the ACTTIV program.

### Program inclusion procedure

Inpatient case managers could discuss program participation with patients during an index inpatient stay at the psychiatric hospital they thought might profit from the transitional intervention. There were no exclusion criteria based on diagnosis or number or duration of inpatient stay(s). If a person wished to participate, the psychiatrist, clinical psychologist or nurse could sign them up for the program. A first meeting would then take place in the clinic and before the discharge^3,20^. All other meetings would then take place after the discharge and in the local community or home of the person.

If a person was asked whether they would like to participate and seemed interested at first but declined during the registration procedure, their case was added to the control group. This setup of the control group was chosen to ensure that treatment and control group were similar regarding the person’s need for transitional support from the case manager’s perspective.

Individuals could also be invited to join outreach programs again if they were readmitted to the psychiatric hospital during the pilot study (April 2019 to March 2022).

### Design

The study’s design is of a naturalistic and non-randomized nature. It includes two groups: the *Intention-to-Treat (ITT) group* with all cases where a person had declared the intention to be treated within the ACTTIV program and started with initial contact and the *control group* with cases where persons had displayed initial interest but then declined program participation. As, from a clinical point of view, patients needed to receive a minimum intervention dose for the treatment to be effective, a *Per-Protocol (PP) group* was defined for further analyses. It included all cases from the ITT group that had utilized a minimum of 6 treatment session days in the ACTTIV program. The dependent variables in our study are described below.

### Outcome measures for evaluation

#### Objective outcome measures

We investigated whether participation in the ACTTIV program was linked to a reduction in early readmissions to the inpatient psychiatric setting (within 6 months after discharge). More specifically, we tested associations with the number of inpatient treatment days, number of inpatient treatment stays, and number of involuntary inpatient admissions. We operationalized all three objective outcome variables as continuous outcome variables and computed them based on the electronic file record of participants in the intervention and control groups. While the investigated period varied in previous research^1,14,21^, we looked at the 183 days (6 months) after discharge from the inpatient setting in the control group and after the start date with the transitional intervention in the intervention group. Looking at the 183 following days, we extracted the number of inpatient treatment days, inpatient treatment stays, and the number of involuntary inpatient admissions to UPK Basel. We chose the 6-month period to increase the likelihood that potential treatment effects were visible^22^ and could be placed in a temporal context with participation in and completion of the ACTTIV program.

#### Subjective outcome measures

Subjective experiences and general satisfaction with program participation were assessed once at the end of the individual ACTTIV program phase. Satisfaction was operationalized as a continuous subjective outcome measure. Whereas unstandardized verbal feedback was exchanged continuously during the 3 months of the program, participants’ general satisfaction was assessed with the Münsterlingen Patient Satisfaction Questionnaire for outpatient settings^23^ (in German: “Münsterlinger Fragebogen zur Patientenzufriedenheit”-ambulant, short: MüPF-ambulant^24^). The questionnaire includes 21 items and assesses both overall satisfaction and satisfaction with different aspects of mental health treatment. Feedback regarding the 21 items is collected with Likert-scales ranging from 1 to 7 with 7 indicating the highest level of satisfaction. Prior to analyzing the results, Item 7 was recoded, as it was the only question that is framed negatively, whereas all other questions are framed positively, meaning higher values represented more satisfaction.

#### Statistical analyses

All analyses reported in this manuscript were computed with R-Studio^25^. To test for differences in diagnoses between conditions using χ^2^ tests, we categorized diagnosis labels into larger groups to warrant a minimum cell size of at least *n* = 5. This resulted in 6 categories (F1X/Mental and behavioral disorders due to psychoactive substance use, F2X/Schizophrenia, schizotypal and delusional disorders, F3X/Mood/affective disorders, F4X/Neurotic, stress-related and somatoform disorders, F6X/Disorders of adult personality and behavior and other, where the latter category included all other diagnoses, the category other, and no information). Given the count-based nature of the objective outcome variables, we computed nonparametric Wilcoxon tests when analyzing differences between intervention and control groups. For additional multiple regression analyses, all objective outcome variables were log-transformed and the control group was used as a reference category.

## Results

The results describe the ACTTIV project team’s composition, program participants (ITT and PP group) versus decliners (control group), and objective as well as subjective outcomes.

### Project team and participants

The ACTTIV program was delivered by a multidisciplinary team consisting of psychiatrists, social workers and nurse practitioners. The project team started with a combination of 225% full-time equivalents (FTE) of nurses (incl. management), 35% FTE of social workers, and 30% FTE of psychiatrists. Step by step, the numbers were increased during the three-year pilot period and the team was expanded to 405% FTE of nurses, 80% FTE of social workers, and 30% FTE of psychiatrists. Aiming at a caseload of about 15 cases per FTE, the ACTTIV team started with 11 cases in the first month (April 2019) but quickly increased the number to 25 and 39 in the two following months. At the end of the pilot phase, the ACTTIV team had 59 concurrent open cases.

During the three-year pilot project, 4605 patients (with 9104 cases) were discharged from the UPK Basel. Overall, 566 patients (with 699 cases) were invited to participate in the ACTTIV program. Any person could (re)join after a readmission to the psychiatric clinic—no matter whether they had participated or declined during a previous stay. This means that in this study design one person could create several cases. During the pilot project, 486 patients (with 595 cases) participated in the ACTTIV program and formed the *intention to treat group* (ITT group) and 103 patients (with 104 cases) declined participation after initial interest and formed the *control group*. Throughout the pilot study period, one participant died after agreeing to join the ITT group but before participating in any treatment sessions. We excluded their data from the analyses below. Data in regards to this topic is lacking for the control group, as contact was limited.

We further investigated the ITT group and the treatment that participants in the ACTTIV program had received. On average, 15.70 (*SD* = 10.75) days with an assertive community treatment appointment had been documented per participant, however, this number ranged from 1 to 67. For 15 participants, no information was documented (which presumably means that they had zero days with appointments). The *per-protocol analysis group* (PP group; cf.^26^ for a similar approach) included 392 patients with 456 cases.

Looking at ICD-10 diagnoses, the biggest group of cases in the program had a main diagnosis of affective disorders (F3X, 31.31% in the ITT group), followed by a main schizophrenia, schizotypal and delusional disorder diagnosis (F2X, 12.63% in the ITT group). The distribution of diagnoses, however, differed significantly between groups; for the ITT vs. control group comparison χ^2^(5) = 27.05, *p* < 0.001 and for the PP vs. control group comparison χ^2^(5) = 20.85, *p* = 0.001. Further details on participants overall and within each group can be found in Table 1.

**Table 1:** Descriptive overview on cases’ gender, age, and diagnosis count in the intention-to-treat (ITT), per-protocol (PP) and control group.

Variable	Overall	ITT group	PP group	Control group
Gender				
Male	283 (40.54%)	231 (38.89%)	169 (37.06%)	52 (50.00%)
Female	391 (56.02%)	344 (57.91%)	274 (60.09%)	47 (45.19%)
No information	24 (3.44%)	19 (3.20%)	13 (2.85%)	5 (4.81%)
Age (years)				
M	51.83	52.21	52.08	49.61
SD	16.71	16.80	16.79	16.10
Diagnosis (ICD 10)				
F0X/Organic, including symptomatic, mental disorders	13 (1.86%)	10 (1.68%)	9 (1.97%)	3 (2.88%)
F1X/Mental and behavioral disorders due to psychoactive substance use	64 (9.17%)	46 (7.74%)	35 (7.68%)	18 (17.31%)
F2X/Schizophrenia, schizotypal and delusional disorders	98 (14.04%)	75 (12.63%)	59 (12.94%)	23 (22.12%)
F3X/Mood/affective disorders	219 (31.38%)	186 (31.31%)	158 (34.65%)	33 (31.73%)
F4X/Neurotic, stress-related and somatoform disorders	76 (10.89%)	63 (10.61%)	56 (12.28%)	13 (12.50%)
F5X/Behavioral syndromes associated with physiological disturbances and physical factors	2 (0.29%)	1 (0.17%)	1 (0.22%)	1 (0.96%)
F6X/Disorders of adult personality and behavior	60 (8.60%)	54 (9.09%)	51 (11.18%)	6 (5.77%)
F8X/Disorders of psychological development	1 (0.14%)	1 (0.17%)	1 (0.22%)	0 (0%)
F9X/Behavioral and emotional disorders with onset usually occurring in childhood and adolescence	3 (0.43%)	3 (0.51%)	3 (0.66%)	0 (0%)
Other/No information	162 (23.21%)	155 (26.09%)	83 (18.20%)	7 (6.73%)

Age was computed as difference in years between cases’ date of birth and the start date of the ACTTIV program for cases in the ITT and PP condition and the discharge date of the index stay for cases in the control condition. The diagnoses represent the main diagnosis coded at the time the patient was included into the intervention or control group.

### Objective outcome measures

Table 2 summarizes the results in regards to the objective outcome measures. When looking at means, all outcome measures were descriptively lower in the PP group compared to the control group. Quantifying this mean difference, the PP group showed 33.57% fewer inpatient treatment days, 35.48% fewer inpatient stays and 44.44% fewer involuntary admissions compared to the control group.

**Table 2:** Descriptive results of outcome variables overall and separately for cases in the per-protocol (PP), intention-to-treat (ITT) and control group.

Statistic	Target variable observed during follow up period of 183 days		
	Number of inpatient treatment days in psychiatric clinic	Number of inpatient stays at the psychiatric clinic	Number of involuntary admissions to the psychiatric clinic
Overall (*n* = 698)			
Range	0–135	0–8	0–3
M (SD)	14.26 (28.52)	0.56 (1.08)	0.08 (0.33)
25% P	0.00	0.00	0.00
Md	0.00	0.00	0.00
75% P	12.00	1.00	0.00
PP group (*n* = 456)			
Range	0–135	0–4	0–3
M (SD)	10.11 (23.34)	0.40 (0.80)	0.05 (0.28)
25% P	0.00	0.00	0.00
Md	0.00	0.00	0.00
75% P	2.00	1.00	0.00
ITT group (*n* = 594)			
Range	0–135	0–7	0–3
M (SD)	14.09 (27.62)	0.55 (1.01)	0.08 (0.33)
25% P	0.00	0.00	0.00
Md	0.00	0.00	0.00
75% P	14.75	1.00	0.00
Control group (*n* = 104)			
Range	0–134	0–8	0–2
M (SD)	15.22 (33.29)	0.62 (1.42)	0.09 (0.34)
25% P	0.00	0.00	0.00
Md	0.00	0.00	0.00
75% P	4.75	1.00	0.00

All 3 outcome variables are count-based, which warrants nonparametric testing. Wilcoxon tests for unpaired groups were therefore computed to compare outcomes between cases in the ITT, PP, and control group. These tests, however, indicated that the observed differences did not reach significance: *W* = 23,204, *p* = 0.657 for the number of inpatient treatment days, *W* = 23,226, *p* = 0.670 for number of inpatient stays, and *W* = 23,151, *p* = 0.310 for the number of involuntary admissions.

Mean differences between the ITT and the control group were smaller. The ITT group showed 7.42% fewer inpatient treatment days, 11.29% fewer inpatient stays and 11.11% fewer involuntary admissions compared to the control group. Comparisons using nonparametric tests were not significant; *W* = 32,287, *p* = 0.370 for the number of inpatient treatment days, *W* = 32,273, *p* = 0.372 for the number of inpatient stays, and *W* = 30,627, *p* = 0.738 for the number of involuntary admissions.

Given the significant differences in distribution of diagnoses between conditions, we further complemented these analyses by computing multiple linear regression models that include condition as an independent variable (reference category: control group) and diagnosis as a control variable (collapsed into 6 categories, see above). Results, however, remain non-significant when investigating the PP and control group (*b* = -0.09, *p* = 0.610 for inpatient treatment days, *b* = -0.06, *p* = 0.252 for inpatient stays, and *b* = -0.01, *p* = 0.495 for involuntary admissions) as well as when investigating the ITT and control group (*b* = 0.13, *p* = 0.495 for inpatient treatment days, *b* = 0.00, *p* = 0.996 for inpatient stays, and *b* = −0.00, *p* = 0.874 for involuntary admissions).

### Subjective outcome measures

Overall, 85 questionnaires were collected after participants completed the ACTTIV program. Assuming that all 562 cases that had been closed during the project phase had received a questionnaire, this would result in an estimated response rate of 15%.

Looking at the feedback, mean values were above 6 for 14 out of 21 items (the Likert-scale ranged from 1 to 7 with 7 indicating the highest level of satisfaction). Mean overall satisfaction (see item 17) was 6.54 (*SD* = 0.99). We also computed a satisfaction score across all items, which indicated a mean satisfaction score of 6.18 (*SD* = 0.82). Using the scale midpoint as a cut-off value, 81 participants indicated positive mean satisfaction (values > 4, averaged across all items), 1 participant was indifferent (at a value of 4), and only 2 participants indicated dissatisfaction to some extent (values < 4; 1 participant had only missing values).

A more detailed picture of participants’ satisfaction with the ACTTIV program can be found in Table 3. The highest positive values were obtained in regards to items 4 and 18, indicating that participants felt treated with respect by the project team and that they would recommend the treatment offer (*M*_*item4*_ = 6.77, *SD* = 0.75; *M*_*item18*_ = 6.60, *SD* = 0.93). The lowest satisfaction values resulted for items regarding the medical treatment, showing that participants were not as satisfied with the explanations regarding potential side effects of medications and with their ability to influence the medical treatment (*M*_*item10*_ = 4.94, *SD* = 2.16; *M*_*item11*_ = 4.98, *SD* = 2.24). The questionnaire also asked for open comments. Participants provided a great deal of positive feedback and described gratitude, for example, for “not having to go through things all by myself in times of a crisis” as well as that the appointments could take place at home. When asking for criticism, participants said that they wished that the program had lasted longer than 3 months and one participant thought that it would be helpful if the transitional treatment would start earlier during the inpatient stay.

**Table 3:** Patient satisfaction after completion of the ACTTIV program.

	Item	*n*	*Min*	*Max*	*Mean*	*SD*
1	The service is easy to reach by telephone	77	2	7	6.47	0.95
2	I was able to adequately explain my situation in the initial consultation	83	1	7	6.31	1.32
3	After joining the program, the next steps were explained to me	81	1	7	6.53	1.00
4	I felt treated with respect by the clinical staff	83	1	7	6.77	0.75
5	When I am in distress, I know where to turn	82	1	7	6.55	1.07
6	I trust the people who are treating me	83	1	7	6.54	1.18
7	I was hesitant to ask questions. (R)	83	1	7	6.11	1.64
8	Treatment goals were agreed with me	79	1	7	5.48	1.80
9	I was able to influence the planning of my treatment	81	1	7	6.04	1.58
10	The effects of the medication and possible side effects were clearly explained to me	54	1	7	4.94	2.16
11	I was able to influence the medication treatment	54	1	7	4.98	2.24
12	The service providers had enough time to talk to me	83	1	7	6.53	1.09
13	I was supported and accompanied in my search for other sources of support (including agencies, self-help groups)	64	1	7	6.38	1.44
14	The collaboration between my relatives and those treating me met my needs	53	1	7	5.83	1.91
15	How helpful did you find working with your caregiver?^a^	78	1	7	6.38	1.21
16	The treatment helped me to better deal with my problems	81	1	7	5.86	1.37
17	Overall, I am satisfied with my treatment	84	1	7	6.54	0.99
18	I would recommend this treatment offer	81	1	7	6.60	0.93
19	I feel well cared for when I have physical problems	56	1	7	5.96	1.46
20	My follow-up care after the assertive community treatment was sufficiently organized	73	1	7	6.33	1.33
21	Since starting treatment with the assertive community treatment program, my condition is now^b^	81	4	7	5.37	0.94

Replies were recorded on a Likert-scale ranging from 1 = *does not apply at all* to 7 = *fully applies*, with an additional option of 8 = *cannot answer* (coded as a missing for the data analysis).

^a^The labels of the Likert scale for this item were 1 = *not helpful at all* to 7 = *very helpful.*

^b^The labels of the Likert scale here were 1 = *much worse* to 7 = *much better.*

As the feedback was collected anonymously, results cannot be further analyzed regarding patients’ diagnoses or the amount and duration of treatment received.

## Discussion

The ACTTIV program is a new treatment offer with the aim to bridge the transition between an inpatient stay at a psychiatric hospital and community living. As early readmission after an inpatient stay is unfortunately rather common, it seems important to support people during this period. The results from ACTTIV’s project phase provide evidence that it was feasible to assemble a multidisciplinary project team and offer transitional and individualized support to a number of people in their homes in the canton of Basel-Stadt in Switzerland. We, however, see that the program may appeal more to a certain group of people: when comparing cases in the intervention and in the control group, the cases differed, for example, in regards to the distribution of diagnoses. Looking at outcome measures, we find that, descriptively, program versus control participants had fewer inpatient days, inpatient stays, and involuntary admissions to psychiatric hospitals during the 6-month follow-up period. For the PP group, for example, we found that inpatient treatment days were 33.57% lower, inpatient stays were 35.48% lower and involuntary admissions were 44.44% lower compared to the control group. However, these differences did not reach statistical significance. Participants’ feedback regarding the ACTTIV program was very positive, both overall and when looking at specific program aspects.

These results from the pilot phase can be carefully interpreted as promising in part: They illustrate the set-up and organization of an important additional mental health treatment that is situated between inpatient and outpatient care and, when integrated into standard care, may broaden the need-centered treatment mix in Switzerland^27^. Furthermore, the first subjective outcomes are positive, showing high levels of satisfaction from ACTTIV participants. Other work also speaks to the positive impact of assertive community treatment on subjective outcomes^26^.

However, in line with previous research reviews^2,3^, our results regarding objective outcomes seem to add to the mixed findings regarding the effectiveness of transitional interventions. Results regarding readmission rates and durations among the intervention group may seem potentially promising, but are not significantly different from the control group.

Drawing comparisons between different programs is, however, difficult. Health care systems differ between countries^28^ and programs need to be tailored to the respective settings and needs of healthcare providers and patients. Different research findings also often vary depending on the outcome in focus. Forchuk et al.^14^, for example, did not find effects of their transitional discharge model on post-discharge costs but when investigating discharge dates in an exploratory fashion, *earlier* discharges were identified for intervention participants in Canada. In Iran, other work found within group differences in hospitalization rates before and after the program and also highlighted that the intervention group had significantly more disease related knowledge^29^. Other work in Switzerland has found null effects for readmission rates and durations, and even showed a decrease in self-reported symptom remissions in the intervention group^30^.

Differences in outcome variables make comparisons challenging, and differences in program elements add to this difficulty: Reynolds et al.^13^, for example, showed lower readmission rates among their experimental group in Scotland, but their treatment additionally included peer support, which may serve as a positive key element. Looking at different time points during a follow up period over 24 months, other work from Germany has found no significant differences between an intervention and control group for their transitional intervention^31^, however, this intervention was deliberately designed to be limited (see p. 186) and only consisted of two sessions.

Some researchers consider this landscape of findings as having no compelling evidence for programs bridging inpatient and community mental health care (see, e.g., the discussion of^30^). Other researchers, however, explicitly state that further data (preferably from randomized-controlled trials) is needed to understand the potential benefits of transitional interventions in more detail and with more certainty^2,32^. This manuscript may aide in this regard by providing additional data from a pilot study on both subjective and objective outcomes. It, nevertheless, also adds to the mix of findings on transitional interventions in the field.

### Limitations

Certain limitations apply in regards to the evaluation study of the ACTTIV program. First, the naturalistic design of the study may contribute to its ecological validity, yet causes challenges when trying to make group comparisons due to selection bias on the level of sampling and allocation to conditions. Inpatient teams decided who might benefit from a transitional intervention and offered it to some but not all individuals. After receiving the suggestion from the inpatient team, program participants *chose* to join the ACTTIV program and were not randomized into an intervention or control arm of the study. Instead, all persons wishing to participate and continue with treatment sessions outside of the clinic formed the ITT/PP groups. The control group was comprised of participants who showed initial interest but did not ultimately join the program. Group sizes were therefore imbalanced, with the control group being much smaller than the ITT/PP groups. While this procedure was selected to ensure that study participants were initially in comparable need of transitional support, it comes with the caveat that treatment and control groups might differ not only in regards to having received the intervention or not, but also in other aspects^33^. Our data already shows significant differences in the distribution of diagnoses. Differences in treatment-related attitudes, expectations, and needs and more general mental health related variables, such as speed of recovery and support outside of the clinic, could be present too. One could speculate that these factors may contribute to the finding that differences in objective outcome measures, such as inpatient treatment days, were not significant, as eventually ITT/PP group participants needed the program support more than the control group who might have had access to other support networks. Moreover, this self-selection could also impact the subjective outcome measures: The response rate was rather low, which again could represent a selection bias, if one speculates that some ACTTIV participants might have been more or less motivated and then also satisfied with the program, and this may impact whether or not they provided feedback.

The structure of our data set further violates the assumption of independence of observations^34^, as the same person could participate or decide against participation in the ACTTIV program multiple times (during every inpatient stay in the pilot project phase), thereby creating several cases within and across conditions. Data on objective outcomes, moreover, only covers stays at the University Psychiatric Clinic Basel, and additional stays in other clinics might occur and could not be considered in this study. However, the UPK Basel is the main service provider of inpatient mental health treatment in the area, so this effect might be limited, especially in severely ill patients with a high risk of rehospitalization.

In order to continue to evaluate the program’s effects, data collection will go on after the first pilot phase. Further evaluations for objective outcomes might entail different analysis methods, such as using survival analyses to test whether program participation is associated with a lower risk of readmission to the psychiatric hospital over time. Additional analysis options would be to look at within-person effects by comparing readmissions after an inpatient stay with and without participating in a bridging offer such as ACTTIV or to use of matching procedures to balance conditions. In regards to subjective outcomes, satisfaction can be monitored continuously and potential changes in self-reported needs at the beginning and at the end of the program may be investigated for all program participants.

In addition to our ideas relating to the data collected as part of the newly established outreach programs, future research is needed to better understand predictors of being offered participation in transitional interventions by health care providers and accepting/rejecting participation by individuals. Future research could also test the effectiveness of transitional interventions using randomized-control trials^35^, limit or statistically control multiple cases of the same participants, access full medical records to assess complete information on objective outcomes, and ideally also compare effectiveness across different healthcare systems. Measures should also be implemented to ensure a high response rate for subjective outcome measures. Our work may provide a stepping-stone in this matter in the context of the health care system of Switzerland, showing that implementing transitional interventions is feasible and seems to be received very well by people after an inpatient stay.

### Outlook and conclusion

Given the ACTTIV program’s successful introduction and first positive outcomes, the pilot phase was extended for another three years until March 2025. The program will be further developed and will, for example, include psychotherapy as an additional treatment offer for participants. The here presented results speak to the program’s feasibility and positive uptake among people after an inpatient stay in the psychiatric hospital. Whereas satisfaction with the transitional intervention was high, differences in hospital readmissions were not statistically different from a control group of people who declined program participation. More research is required to test for causal effects of transitional interventions, however, ACTTIV already represents a guideline aligned step to offer more need-oriented and individualized treatment supporting the transition into community living.

## Data Availability

Data availability statement The data is not publicly available but an anonymized version of the here presented variables can be shared upon request (contact: versorgungsforschung@upk.ch).
